# Runs of Homozygosity Islands in Autochthonous Spanish Cattle Breeds

**DOI:** 10.3390/genes15111477

**Published:** 2024-11-15

**Authors:** C. Hervás-Rivero, N. Mejuto-Vázquez, D. López-Carbonell, J. Altarriba, C. Diaz, A. Molina, R. Rodríguez-Bermúdez, J. Piedrafita, J. A. Baro, L. Varona

**Affiliations:** 1 Instituto Agroalimentario de Aragón (IA2), Departamento de Anatomía, Embriología y Genética, Facultad de Veterinaria, Universidad de Zaragoza, C. Miguel Servet, 177, 50013 Zaragoza, Spain; chervas@unizar.es (C.H.-R.); davidlc@unizar.es (D.L.-C.); altarrib@unizar.es (J.A.); 2 Departamento de Anatomía, Producción Animal y Ciencias Clínicas Veterinarias, Facultad de Veterinaria, Universidad de Santiago de Compostela, Av. Carvallo Calero, 27002 Lugo, Spain; nicolas.mejuto.vazquez@usc.es (N.M.-V.); ruth.rodriguez@usc.es (R.R.-B.); 3 Departamento de Mejora Genética Animal, Instituto Nacional de Investigación y Tecnología Agraria y Alimentaria (INIA-CSIC), 28040 Madrid, Spain; 4 Departamento de Genética, Facultad de Veterinaria, Universidad de Córdoba, 14071 Córdoba, Spain; ge1moala@uco.es; 5 Departamento de Ciencia Animal y de los Alimentos, Facultat de Veterinaria, Universitat Autònoma de Barcelona, Bellaterra, 08193 Barcelona, Spain; jesus.piedrafita@uab.cat; 6 Departamento de Ciencias Agroforestales, ETS de Ingenierías Agrarias, Universidad de Valladolid, 34004 Palencia, Spain; jesusangel.baro@uva.es

**Keywords:** autochthonous cattle, runs of homozygosity (ROH), ROH islands, selection signatures

## Abstract

Background/Objectives: Understanding the genetic architecture of autochthonous European cattle breeds is important for developing effective conservation strategies and sustainable breeding programs. Spanish beef cattle, which trace their origins to ancient migrations from the Near East with later admixture from African populations, exhibit a rich genetic diversity shaped by environmental adaptation and selective breeding. Runs of Homozygosity (ROH) are extended stretches of identical genetic material inherited from both parents. They serve as indicators of inbreeding and selection signatures within populations. ROH islands, or regions of the genome where ROH segments are highly concentrated across individuals within a breed, indicate genomic regions under selective pressure. Methods: This study explores the distribution of ROH islands across seven Spanish beef cattle breeds (Asturiana de los Valles, Avileña-Negra Ibérica, Bruna dels Pirineus, Morucha, Retinta, Pirenaica, and Rubia Gallega). By analyzing high-density SNP data, we characterized ROH patterns and identified genomic regions with high levels of homozygosity, which may indicate selection pressures or common ancestry. Results: Our findings revealed breed-specific ROH patterns as well as shared ROH islands, underscoring genetic relationships and differentiation among the breeds. Notably, Morucha displayed the highest number of ROH, while Asturiana de los Valles had the fewest. F_ROH_ values, which indicate genomic inbreeding, varied among the breeds, with Morucha and Retinta being associated with higher values. We identified 57 ROH islands, with shared regions among populations that suggest common ancestral selection pressures. Key genes within these regions, like MSTN, are associated with muscle growth, body weight, and fertility. Conclusions: This study offers valuable insights for breeding strategies and conservation efforts, highlighting the genetic diversity and historical background of Spanish cattle breeds.

## 1. Introduction

The formation of autochthonous cattle breeds in Europe, including Spanish breeds, can be traced back to the migration of cattle from the Near East, followed by crossbreeding with African cattle [1,2]. Initially used as triple-purpose animals (draft work, milk, and meat), these breeds gradually diverged into present-day breeds, influenced by natural selection processes driven by environmental conditions, management practices, and geographical boundaries. More recent differentiation of breeds was driven by phenomena like isolation, genetic drift, adaptation to specific environments, and the systematic application of modern breeding techniques [3].

Runs of homozygosity (ROH) are contiguous segments of the genome where an individual inherits identical haplotypes from both parents [4]. These regions of homozygosity have emerged as valuable indicators of inbreeding and potential signatures of selection within populations across various species. Similarly, ROH islands are specific regions of the genome that display pronounced ROH patterns shared among individuals within or across populations [5,6]. They have attracted interest due to their potential biological significance and implications for genetic research. The presence of ROH islands within populations suggests underlying genetic phenomena, such as recent common ancestry or selection pressures leading to increased homozygosity in particular genomic regions [7]. Understanding the distribution and characteristics of ROH islands is helpful for deciphering population histories, identifying regions under selection, and informing breeding strategies in animal genetics.

This study aims to provide an overview of ROH islands across a diverse group of Spanish beef cattle populations that are closely related [8,9]. These breeds are Asturiana de los Valles (AV), Avileña—Negra Ibérica (ANI), Bruna dels Pirineus (BP), Morucha (Mo), Pirenaica (Pi), Retinta (Re), and Rubia Gallega (RG). The methodology employed involved high-density SNP data obtained from a standardized bovine genotyping array.

## 2. Materials and Methods

### 2.1. Data and Data Preparation

We used genotype data from the Illumina BovineHD BeadChip (777 K) (Illumina Inc., San Diego, CA, USA) from seven autochthonous Spanish cattle breeds. The dataset comprised 336 unrelated individuals (50 AV, 48 ANI, 50 BP, 50 Mo, 48 Pi, 46 Re, and 44 RG), with an equal number of males and females. The animals were chosen from different and separated geographical areas, taking care to avoid known relationships. Quality control of the genotype data was conducted using PLINK v1.9 [10], with parameters set for missing genotype rate (*--geno*) at 0.1 and missing rate per individual (*--mind*) also at 0.1, resulting in 704277 markers remaining. No individuals were excluded, and the overall genotyping rate was 99.86%. In line with the study of Ferenčaković et al. [8], we did not apply a minor allele frequency filter, as removing these SNPs could lead to the loss of significant information for the estimation of runs of homozygosity (ROH). 

### 2.2. ROH Calculation

ROH were detected using the consecutiveRUNS function from the detectRUNS [9] package for R software 4.4.1 [10]. This function employs the method developed by Marras et al. [11]. Following the approach of Ferenčaković et al. [12], we categorized ROH into five groups based on their base-pair length (1 to 2 Mb, 2 to 4 Mb, 4 to 8 Mb, 8 to 16 Mb, >16 Mb). Different strategies were used for calculations based on these categories. Initially, we calculated all potential ROH to determine the average base-pair length of each group. Using these measurements, the allowed number of heterozygotes was calculated by multiplying the average length by the assumed genotype error rate of 0.25%. The numbers of missing SNPs allowed in an ROH were determined using the average percentage of missing genotypes, which is 0.14%. The final parameters for each group were set as follows: for lengths of 1 to 2 Mb, 2 to 4 Mb, 4 to 8 Mb, 8 to 16 Mb, and greater than 16 Mb, the allowed number of missing genotypes was 1, 1, 2, 4, and 8, respectively, while the allowed number of heterozygotes was 1, 2, 4, 7, and 12, respectively. The minimum SNP length for an ROH was set at 15, and the maximum gap between SNPs was set at 100,000 bp, in accordance with Ferenčaković et al. [8]. Once these parameters were calculated, they were used to recalculate ROH according to them.

### 2.3. F_ROH_ Calculation

F_ROH_ was used as a measure of genomic inbreeding. It represents the proportion of the genome that is encompassed in ROH [13] and is calculated by dividing the sum of all ROH lengths in an individual by the total length of the genome.

### 2.4. Identification of ROH Islands

A custom R script was developed to create a presence-absence matrix of SNPs within the ROH for each animal. This approach allowed us to determine the percentage of animals in each population that share the same SNP within an ROH. Shared ROH regions are defined as genomic areas with reduced genetic diversity, characterized by high homozygosity around specific sites, which can help to identify targets of positive and intense selective pressure [14]. For each population, we established a threshold of shared SNPs in ROH to define ROH islands. These thresholds were determined using standard normal z-scores calculated from the distribution of SNPs in ROH. SNPs falling within the bottom 0.001% of *p*-values according to a z-score table for ROH incidence were identified as forming ROH islands, adapting the methodology described by Purfield et al. [7] and Gorssen et al. [15] to the size of the genomic chip used in this study. 

### 2.5. Identification of Candidate Genes

The reference cow genome UMD2.1 was utilized with the ENSEMBL BIOMART tool (release 110) to identify genes located within the ROH island regions.

## 3. Results

### 3.1. ROH Analysis

A total of 20,522 ROH were identified across all populations, with an average of 923.8 SNPs and an average base-pair length of 3,481,254 bp. The population with the highest number of ROH was Mo (4415), while the maximum average size in both SNP count and length was found in Re and ANI. Conversely, AV had the fewest ROH, and BP exhibited the smallest ROH size. A summary of all the ROH by population can be found in Table 1.

**Figure 1:**
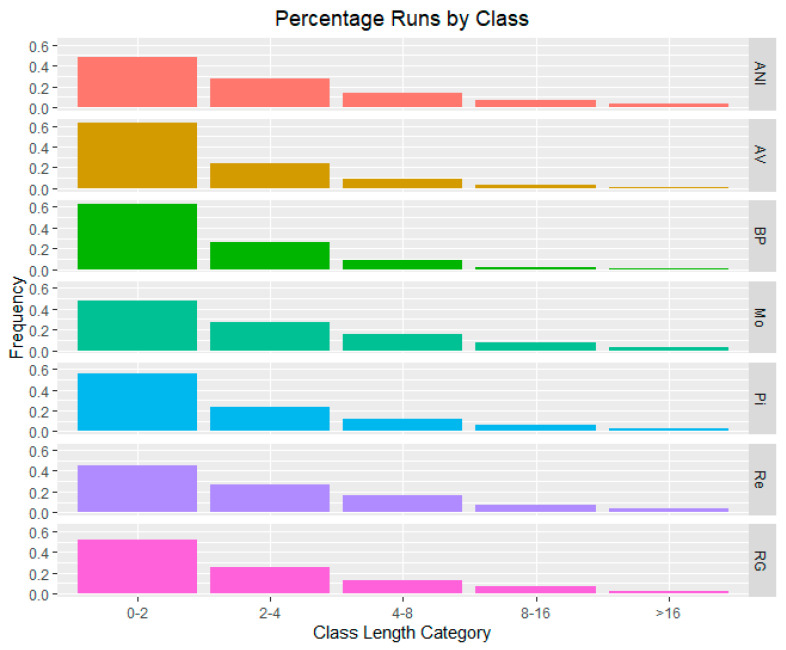
Percentage of runs of homozygosity by class size (0–2 Mb, 2–4 Mb, 4–8 Mb, 8–16 Mb, >16 Mb) for Avileña-Negra Ibérica (ANI), Asturiana de los Valles (AV), Bruna dels Pireneus (BP), Morucha (Mo), Pirenaica (Pi), Retinta (Re), and Rubia Gallega (RG) populations.

The percentage of ROH by class is illustrated in Figure 1. This figure shows a higher frequency of ROH smaller than 2 Mb, which account for over 60% of the total ROH in BP. The other populations exhibit frequencies greater than 40%. In contrast, the population with the largest percentage of ROH exceeding 16 Mb is ANI, confirming its greater average ROH length.

### 3.2. F_ROH_ Results

**Figure 2:**
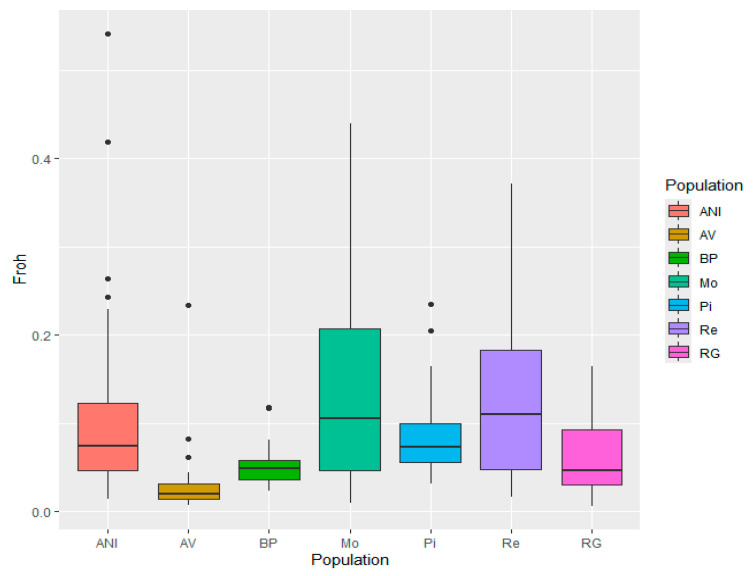
Box and whisker plot of F_ROH_ for Avileña-Negra Ibérica (ANI), Asturiana de los Valles (AV), Bruna dels Pireneus (BP), Morucha (Mo), Pirenaica (Pi), Retinta (Re), and Rubia Gallega (RG) populations.

The results of F_ROH_ are presented in Figure 2. The average F_ROH_ decreased from 0.12 to 0.02. According to these results, AV and BP exhibited the lowest levels of inbreeding, with the smallest variation. Conversely, Mo and Re had the highest average F_ROH_, along with greater variability among individuals, which aligns with the lower estimates of effective population size for these populations from a previous study [16]. 

### 3.3. ROH Islands

**Figure 3:**
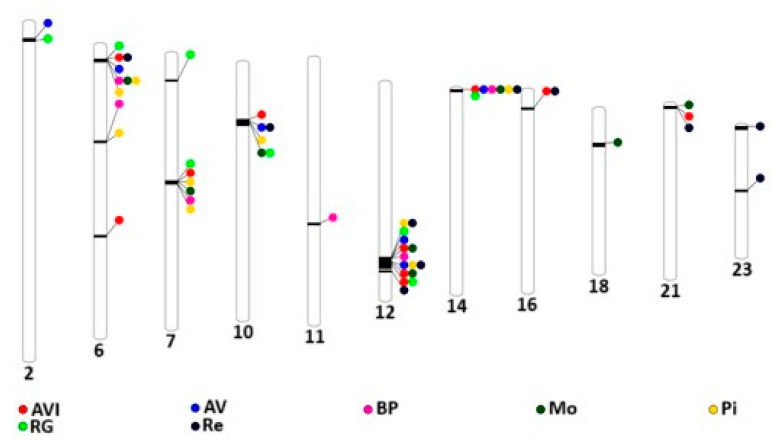
Representation of ROH islands in cattle chromosomes where they are located for Avileña-Negra Ibérica (ANI), Asturiana de los Valles (AV), Bruna dels Pireneus (BP), Morucha (Mo), Pirenaica (Pi), Rubia Gallega (RG), and Retinta (Re) populations.

The procedure to define the thresholds for the purpose of identifying ROH islands revealed that the minimum percentages of shared SNPs in ROH for each population were 34.7% for AV, 35.4% for ANI, 62.0% for BP, 44.0% for Mo, 41.6% for Pi, 34.7% for Re, and 43.1% for RG. The genomic scans for the percentage of individuals within ROH are presented in Supplementary Figure S1. A total of 57 regions were identified as ROH islands (10 in AV, 6 in ANI, 6 in BP, 8 in Mo, 9 in Pi, 10 in Re, and 8 in RG). The distribution of ROH islands across the autosomal genome was heterogeneous, with locations on chromosomes 2, 6, 7, 10, 11, 12, 14, 16, 18, 21, and 23, as illustrated in Figure 3 and Table 2, which also identifies the candidate genes located within these regions.

## 4. Discussion

ROH islands are a widely used and accepted method that has been applied in studies of various cattle populations [17,18,19], and in other livestock species [15] to compare selection signatures and the genetic proximity of different populations. Additionally, the study of F_ROH_ is well established for estimating inbreeding and distinguishing between ancient or modern inbreeding [20]. 

Our results showed a higher percentage of small ROH, indicating that ancient inbreeding is more relevant than recent inbreeding. This implies that the selection breeding programs are not contributing significantly to inbreeding and that the effective population size is sufficiently large to maintain genetic diversity [16]. However, our F_ROH_ results showed higher inbreeding than a pedigree-based study [21,22], which can be explained by the limited depth of the available pedigree for some of this populations [22]. 

The ROH island shared among populations agree with the findings of Cañas-Alvarez et al. [3], who studied the genetic diversity across these autochthonous breeds. Their work highlighted the distinctiveness of the Pi breed and the genetic proximity between AV and RG, as well as between ANI, Mo, and Re. In our study, AV and RG, Re, Mo and ANI, and Pi and BP shared specific regions. Furthermore, the more divergent populations (Re, AV, and RG) exhibited unique ROH islands. There were six genomic regions shared across all the populations. However, most of these shared ROH islands have not been identified in other cattle populations, suggesting that they likely originate from a common ancestor from which these populations diverged. 

### Candidate Genes

A total of 183 genes were found in the candidate regions (Supplementary Table S1). Six regions were shared across all populations and are located on BTA6, BTA7, BTA10, BTA12, and BTA14. In the genomic region on BTA6 (6: 5,207,637–6,694,159), several notable genes are present, including PDE5A (*Phosphodiesterase 5A*), FABP2 (*Fatty Acid Binding Protein 2*), and MYOZ2 (*Myozenin 2*) genes. The PDE5A gene encodes a phosphodiesterase that is highly expressed in the testis and may contribute to testicular tissue alterations, such as decreased testis weight, degeneration, and atrophy of the seminiferous epithelium, underscoring its role in reproductive health [23]. The FABP2 gene is thought to act as a lipid-sensing component essential for energy homeostasis, highlighting its importance in metabolic regulation [24]. Meanwhile, the MYOZ2 gene plays a role in muscle growth and development [25].

The genomic region of BTA7 (7: 51,157,314–52,068,636) includes several important genes. The NME5 (*NME/NM23 Family Member 5*) gene is associated with body weight and immunity in cattle [26]. The SLBP2 (*Stem-Loop Histone MRNA Binding Protein*) gene is implicated in oocyte characterization [27]. The KDM3B (*Lysine Demethylase 3B*), a histone H3 demethylase, plays a crucial role in spermatogenesis and normal male sexual behavior, and has been also identified as a fertility-related candidate gene in sheep [28]. Additionally, the EGR1 (*Early Growth Response 1*) gene has various functions across multiple contexts, including the regulation of differentiation and growth development [29]. 

In the ROH island located on BTA10 (10: 22,525,115–25,399,206), we were unable to identify any notable candidate genes. In contrast, BTA12 contains two closely linked ROH islands (12: 70,348,202–72,147,564 and 12: 72,400,144–76,710,313). Within these regions, we found several potential candidate genes, including DNAJC3 (*DnaJ Heat Shock Protein Family Member C3*), which is associated with embryonic development in zebrafish [30], and whose knockout mice exhibit a diabetic phenotype [31], highlighting its role in both development and metabolic disorders. Additionally, these regions contain the OXGR1 (*Oxoglutarate Receptor 1*) gene, which encodes a G protein-coupled receptor (GPCR) involved in various inflammatory disorders [32]. The FARP1 (*FERM*, *ARH*/*RhoGEF*, and *pleckstrin domain protein 1*) gene is implicated in milk quality by regulating milk urea nitrogen [33]. The UBAC2 (*UBA Domain Containing 2*) gene produces transcripts that play a significant role in the ubiquitin–proteasome system, which is important for muscle loss [34]. Lastly, the CLYBL (*Citramalyl-CoA Lyase*) gene has been shown to be differentially expressed in cows with varying milk citrate contents, suggesting its role in milk composition [35].

The last ROH island shared by the seven populations (14: 74,984–1,226,863) contains several noteworthy genes, including FOXH1 (*forkhead box H1*) and PPP1R16A (*protein phosphatase 1 regulatory subunit 16A*), both identified as candidate genes influencing milk saturated fatty acid (SFA)0 and C16:0 content in Holstein cows [36,37]. Additionally, this region includes the DGAT1 (*Diacylglycerol O-Acyltransferase 1*) gene, which is a major gene controlling milk fat content [38]. TONSL (*Tonsoku Like, DNA Repair Protein*) is recognized as a candidate gene affecting milk traits in cattle through genotype-by-sequencing association studies [39]. The VPS28 (*VPS28 Subunit Of ESCRT-I*) gene is implicated in milk fat regulation due to its functional role in bovine mammary epithelial cells [40]. CPSF1 (*Cleavage And Polyadenylation Specific Factor 1*) is involved in the final step of triacylglycerol synthesis, significantly impacting milk production traits [41]. GRINA (*Glutamate Ionotropic Receptor NMDA Type Subunit Associated Protein 1*) is also linked to milk yield and fat content in Holstein cows [42]. The last gene located in this region was EEF1D (*Eukaryotic Translation Elongation Factor 1 Delta*), which shows a strong association with overall milk production traits [43]. 

Several regions are shared among subsets of populations. RG y AV are double-muscled populations [44,45] and, notably, share an ROH island on BTA2 (2: 6,177,012–7,767,238) that contains the MSTN (*Myostatin*) gene, which is associated with the double-muscle phenotype [46]. These results are consistent with the identification of ROH islands in the same region in other cattle populations where double-muscling is present, such as Limousin [15,19], Piemontese [11], and Aubrac [15]. 

Three populations (Mo, ANI, and Re) share an ROH island at 21: 530,964–1,794,327, which has been also identified in Simmental [47] and in several Italian beef cattle breeds [48]. This region contains the *MKRN3* (*Makorin Ring Finger Protein 3*), *MAGEL2* (*MAGE Family Member L2*) and *NDN* (*Necdin, MAGE Family Member*) genes. These genes are important for the epigenetic regulation of precocious puberty onset, reproductive hormone synthesis, oocyte development, and embryo implantation in both cattle and humans, as well as for compatibility in the Aosta breed [49,50,51]. 

Moreover, the ROH island shared by BP and Pi populations (6: 38,429,780–39,461,621) included genes like LAP3 (*Leucine Aminopeptidase 3*), which may greatly benefit the simultaneous improvement of mastitis resistance and milk yield traits in dairy cattle [52], and MED28 (*Mediator Complex Subunit 28*), a gene involved in the regulation of cell proliferation and cycle [53]. LCORL (*Ligand Dependent Nuclear Receptor Corepressor Like*) and DCAF16 (*DDB1 And CUL4 Associated Factor 16*) were also identified for bone weight, and these two genes have been captured as the candidate genes for calving ease [54,55,56]. This genomic region has been associated with ROH islands across a wide range of populations, including Reggiana [17], Pinzgau [18], Simmental [19], Charolais [19], Montbeliarde [19], Alpine Grey [57], and Tyrol Grey [58].

Both the ANI and Re populations also share a region (16: 6695203–7749635) that contains KCNT2 (*Potassium Sodium-Activated Channel Subfamily T Member 2*), which is associated with endometritis occurring within 150 days after calving in first-parity Canadian Holstein cows, and is a primary candidate gene for ketosis in dairy cattle [59,60].

In addition, there are ROH islands present exclusively in specific populations (ANI, BP, Mo, and Re). The ANI population features an ROH island on BTA6 (6: 76,883,785–77,963,194), which includes the ADGRL3 (*Adhesion G Protein-Coupled Receptor L3*) gene, which is associated with protein yield and percentage in Holsteins [61]. The BP population has an ROH island on BTA11 (11: 66,466,242–67,445,456) that contains the PPP3R1 (*Protein Phosphatase 3 Regulatory Subunit B*, *α*) gene, identified as a candidate gene linked with muscle content and ribeye area [62]. Additionally, this region includes ARHGAP25 (*Rho GTPase Activating Protein 25*), which is associated with Rac1 function and plays a role in bone health [63], as well as the *BMP10* (*Bone Morphogenetic Protein 10*) gene, which affects angiogenesis, emphasizing its significance in vascular development and function [64]. The genetic origin of the BP population is an admixture of ancient Brown Swiss with local Catalonian populations, and this genomic region has been associated with ROH islands in other Brown Swiss-related populations [65,66].

A unique region (18: 13,372,279–15,023,735) was found in the Mo population, where several notable genes such as CA5A (*Carbonic Anhydrase 5A*), MVD (*Mevalonate Diphosphate Decarboxylase*), SPATA2L (*Spermatogenesis Associated 2 Like*), FANCA (*FA Complementation Group A*), and MC1R (*Melanocortin 1 Receptor*) were found. The CA5A gene has been implicated in fertility in Holstein cattle [67] and the MVD gene has been identified in selective sweeps for adaptation and productivity across different cattle breeds [68,69]. Further, the SPATA2L gene is crucial for sperm formation fertility [70], and the FANCA is vital for the maintenance of folliculogenesis [71]. Finally, the MC1R gene has been associated with strong selection signatures in cattle, indicating its role in adaptive traits [72,73,74], and has been linked to ROH islands in Holstein [19], Polish Red [19], Marchigiana [11], and Marismeña [15].

Lastly, the Re population also had specific regions in BTA23 (23: 14,911–1,506,210 and 23: 25,642,674–26,729,844). This latter genomic region was associated with an ROH island in the Cinisara breed [17], and contains the KHDRBS2 (*KH RNA Binding Domain Containing, Signal Transduction Associated 2*) gene, associated with reproduction traits in Bohai Black Cattle [75]. 

## 5. Conclusions

This study provided a comprehensive analysis of ROH island distribution across seven autochthonous Spanish beef cattle breeds, offering insights into their origins, genetic diversity, and breeding practices. By investigating runs of homozygosity (ROH) and ROH islands, the study elucidated patterns of ancient consanguinity and modern inbreeding within these breeds. The findings highlighted distinct ROH patterns among the breeds, with variations in both the number and size of ROH, reflecting historical selection pressures and modern breeding practices. The analysis of the genomic ROH (F_ROH_) values further underscored varying levels of inbreeding across populations. Further, the identification of ROH islands and shared genomic regions unveiled common ancestral selection pressures and genetic relationships among the breeds. Finally, key genes within these regions provided insights into traits related to muscle growth, body weight, and fertility, informing future breeding strategies. 

## Figures and Tables

**Table 1:** Statistics of runs of homozygosity (ROH) number and size in the Avileña-Negra Ibérica (ANI), Asturiana de los Valles (AV), Bruna dels Pirineus (BP), Morucha (Mo), Pirenaica (Pi), Rubia Gallega (RG), and Retinta (Re) breeds.

Breed	N_ROH	A_ROH ± SD	N_SNP + SD	MaxSNP	MeanL + SD	MaxL
ANI	3314	69.0 ± 32.3	1067.1 ± 1554.3	18079	3.9 ± 5.4	66.6
AV	1364	27.3 ± 13.3	608.7 ± 887.0	9083	2.6 ± 3.1	35.2
BP	2708	54.16 ± 11.3	585.5 ± 632.8	16562	2.3 ± 2.1	59.6
Mo	4415	88.3 ± 49.6	996.8 ± 1283.6	16562	3.7 ± 4.6	59.6
Pi	2952	61.5 ± 17.5	912.9 ± 1268.7	14256	3.4 ± 4.5	49.4
RG	1920	43.63 ± 17.5	913.0 ± 1210.6	10832	3.5 ± 4.2	38.0
Re	3849	77.0 ± 48.7	1080.1 ± 1432.3	16684	3.9 ± 5.1	60.0

N_ROH: number of ROH; A_ROH: average number of ROH per individual; N_SNP: average number of SNP; MaxSNP: maximum number of SNP per ROH; MeanL: average number of Mb per ROH; maxL: maximum number of Mb per ROH.

**Table 2:** Chromosome (BTA), base-pair number of start (Start), base-pair number of end (End), population and number of SNP (nSNP) for the identified ROH islands.

BTA	Start	End	Population	nSNP
2	6,177,012	7,767,238	AV, RG	454
6	5,207,637	6,694,159	All	61
6	38,429,780	39,461,621	BP, Pi	270
6	76,883,785	77,963,194	ANI	291
7	10,192,273	10,969,214	RG	19
7	51,157,314	52,068,636	All	186
10	22,525,115	25,399,206	All	34
11	66,466,242	67,445,456	BP	260
12	70,348,202	72,147,564	All	20
12	72,400,144	76,710,313	All	61
14	74,984	1,226,863	All	28
16	6,695,203	7,749,635	ANI, Re	22
18	13,372,279	15,023,735	Mo	364
21	530,964	1,794,327	Mo, ANI, RE	100
23	14,911	1,506,210	Re	228
23	25,642,674	26,729,844	Re	14

## Data Availability

The original data presented in the study are openly available in Zenodo public repository with DOI https://doi.org/10.5281/zenodo.58595.

## Supplementary Materials

The following supporting information can be downloaded at: https://www.mdpi.com/article/10.3390/genes15111477/s1, Figure S1: Genomic scan for the percentage of individuals within ROH; Table S1: List of genes located within the ROH islands.
