# Supplementary material for: Runs of Homozygosity Islands in Autochthonous Spanish Cattle Breeds
Source: Genes (Basel). 2024 Nov 15;15(11):1477. doi: 10.3390/genes15111477 (PMC11593383; doi:10.3390/genes15111477)

Supplementary Figure S1. Genomic Scan of the Percentage of Individuals within Runs of Homozygosity.

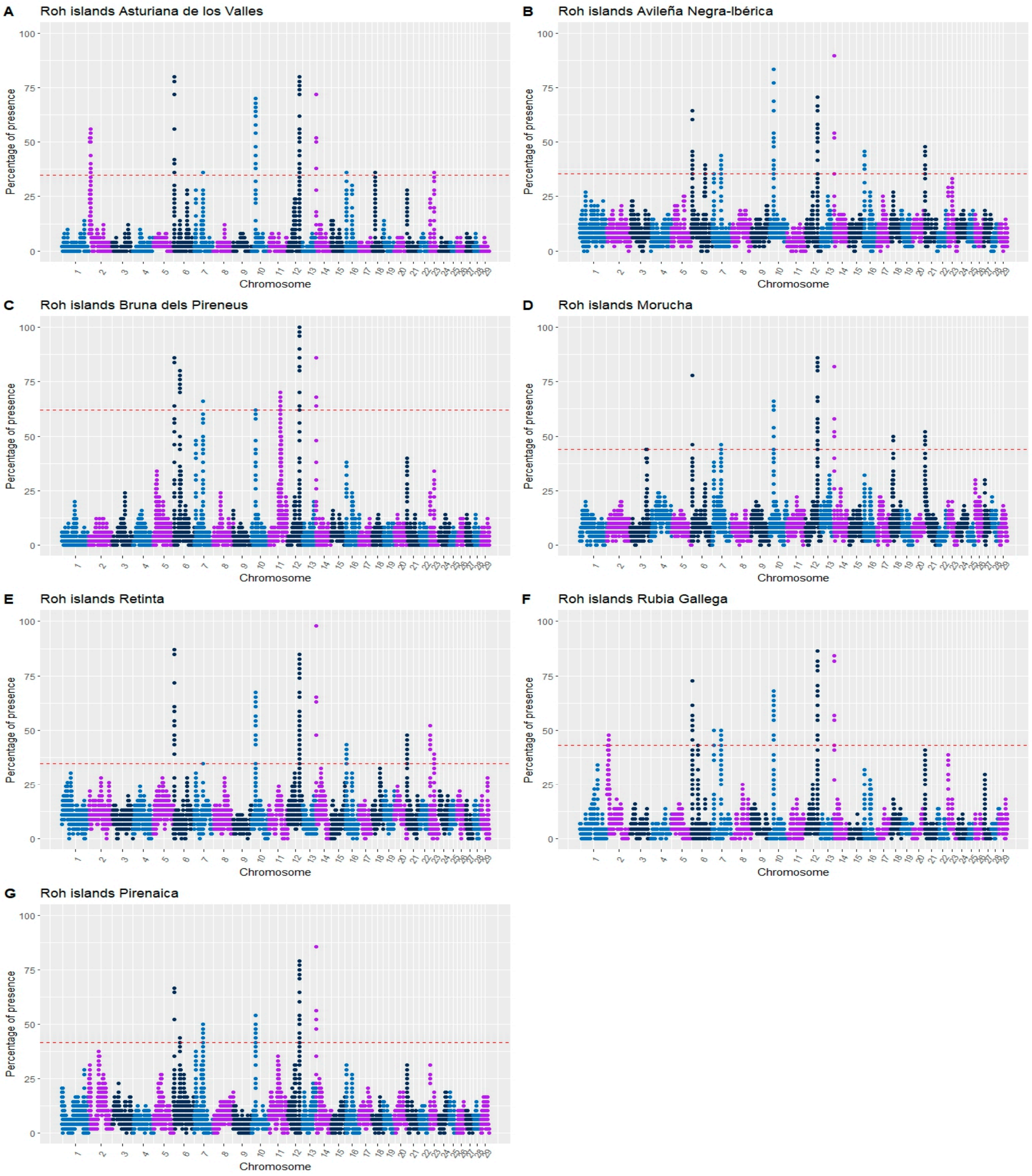

Supplement: Supplementary file 1 [file genes-15-01477-s001.zip › genes-3305973-supplementary/Supplementary Figure.pdf]
