# Supplementary material for: Runs of Homozygosity Islands in Autochthonous Spanish Cattle Breeds
Source: Genes (Basel). 2024 Nov 15;15(11):1477. doi: 10.3390/genes15111477 (PMC11593383; doi:10.3390/genes15111477)
Supplement: Supplementary file 1 [file genes-15-01477-s001.zip › genes-3305973-supplementary/Supplementary Table.pdf]

Supplementary Table S1. List of genes located within Runs of Homozygosity islands.

Gen	Gen name	CHR	Start	End
MSTN	*Myostatin*	2	6278630	6285486
PMS1	*PMS1 Homolog 1, Mismatch Repair System Component*	2	6460095	6592355
ORMDL1	*ORMDL Sphingolipid Biosynthesis Regulator 1*	2	6572135	6584583
OSGEPL1	*O-Sialoglycoprotein Endopeptidase Like 1*	2	6592659	6603565
ANKAR	*Ankyrin And Armadillo Repeat Containing*	2	6605784	6657432
ASNSD1	*Asparagine Synthetase Domain Containing 1*	2	6665574	6676526
SLC40A1	*Solute Carrier Family 40 Member 1*	2	6776416	6800154
WDR75	*WD Repeat Domain 75*	2	6935086	6976756
COL5A2	*Collagen Type V Alpha 2 Chain*	2	7198509	7357287
COL3A1	*Collagen Type III Alpha 1 Chain*	2	7375289	7414912
PDE5A	*Phosphodiesterase 5A*	6	5745779	5894011
FABP2	*Fatty Acid Binding Protein 2*	6	5970114	5973346
USP53	*Ubiquitin Specific Peptidase 53*	6	6014820	6054142
MYOZ2	*Myozenin 2*	6	6097581	6135997
SYNPO2	*Synaptopodin 2*	6	6233764	6435034
SEC24D	*SEC24 Homolog D, COPII Coat Complex Component*	6	6491225	6606147
METTL14	*Methyltransferase 14, N6-Adenosine-Methyltransferase Non-Catalytic Subunit*	6	6640184	6679178
LAP3	*Leucine Aminopeptidase 3*	6	37140752	37166191
MED28	*Mediator Complex Subunit 28*	6	37169216	37175026
FAM184B	*Family With Sequence Similarity 184 Member B*	6	37181075	37301540
HCAP-G	*Non-SMC Condensin I Complex Subunit G*	6	37277323	37378124
DCAF16	*DDB1 And CUL4 Associated Factor 16*	6	37315442	37320955
LCORL	*Ligand Dependent Nuclear Receptor Corepressor Like*	6	37380296	37557106
ADGRL3	*Adhesion G Protein-Coupled Receptor L3*	6	77075369	77651502
NME5	*NME/NM23 Family Member 5*	7	49553095	49581666
BRD8	*Bromodomain Containing 8*	7	49592340	49612744
KIF20A	*Kinesin Family Member 20A*	7	49612809	49642263
CDC23	*Cell Division Cycle 23*	7	49613257	49621657
GFRA3	*GDNF Family Receptor Alpha 3*	7	49676538	49695901
CDC25C	*Cell Division Cycle 25C*	7	49701226	49731890
SLBP2	*Stem-Loop Histone MRNA Binding Protein*	7	49732269	49736500
FAM53C	*Family With Sequence Similarity 53 Member C*	7	49736828	49746610
KDM3B	*Lysine Demethylase 3B*	7	49749644	49805938
REEP2	*Receptor Accessory Protein 2*	7	49808753	49814912

TRAV24	*T Cell Receptor Alpha Variable 24*	10	23697580	23720368
TRAV16	*T Cell Receptor Alpha Variable 16*	10	25060218	25061519
C1D	*C1D Nuclear Receptor Corepressor*	11	66493584	66514419
WDR92	*Dynein Axonemal Assembly Factor 10*	11	66560111	66589114
PNO1	*Partner Of NOB1 Homolog*	11	66589551	66598955
PPP3R1	*Protein Phosphatase 3 Regulatory Subunit B, Alpha*	11	66604007	66667996
CNRIP1	*Cannabinoid Receptor Interacting Protein 1*	11	66705245	66729498
PLEK	*Pleckstrin*	11	66786975	66814889
FBXO48	*F-Box Protein 48*	11	66861857	66865204
APLF	*Aprataxin And PNKP Like Factor*	11	66865277	66958571
PROKR1	*Prokineticin Receptor 1*	11	67001028	67025387
ARHGAP25	*Rho GTPase Activating Protein 25*	11	67091343	67183466
BMP10	*Bone Morphogenetic Protein 10*	11	67211523	67219184
GKN2	*Gastrokine 2*	11	67286584	67291599
FOV	*Gastrokine 1*	11	67308836	67314682
ANTXR1	*ANTXR Cell Adhesion Molecule 1*	11	67359790	67615063
CLDN10	*Claudin 10*	12	72904593	73009466
DZIP1	*DAZ Interacting Zinc Finger Protein 1*	12	73010630	73060581
DNAJC3	*DnaJ Heat Shock Protein Family (Hsp40) Member C3*	12	73095336	73160534
UGGT2	*UDP-Glucose Glycoprotein Glucosyltransferase 2*	12	73169073	73313726
HS6ST3	*Heparan Sulfate 6-O-Sulfotransferase 3*	12	73363796	74075781
OXGR1	*Oxoglutarate Receptor 1*	12	74192308	74193321
MBNL2	*Muscleblind Like Splicing Regulator 2*	12	74410787	74571445
RAP2A	*RAP2A, Member Of RAS Oncogene Family*	12	74632896	74673056
IPO5	*Importin 5*	12	75029683	75069614
FARP1	*FERM, ARH/RhoGEF And Pleckstrin Domain Protein 1*	12	75180883	75458745
STK24	*Serine/Threonine Kinase 24*	12	75461372	75554716
SLC15A1	*Solute Carrier Family 15 Member 1*	12	75634639	75662955
DOCK9	*Dedicator Of Cytokinesis 9*	12	75720835	75894298
UBAC2	*UBA Domain Containing 2*	12	76079653	76300630
GPR18	*G Protein-Coupled Receptor 18*	12	76118033	76121780
GPR183	*G Protein-Coupled Receptor 183*	12	76165404	76180037
TM9SF2	*Transmembrane 9 Superfamily Member 2*	12	76343402	76394962
CLYBL	*Citramalyl-CoA Lyase*	12	76430686	76660289
ZNF250	*Zinc Finger Protein 250*	14	239034	247917
ZNF16	*Zinc Finger Protein 16*	14	261621	275771
ZNF34	*Zinc Finger Protein 34*	14	309282	313478
RPL8	*Ribosomal Protein L8*	14	321231	324177
ZNF7	*Zinc Finger Protein 7*	14	332701	339111
COMMD5	*COMM Domain Containing 5*	14	347689	349724

ARHGAP39	*Rho GTPase Activating Protein 39*	14	359395	414811
LRRC24	*Leucine Rich Repeat Containing 24*	14	417616	424777
LRRC14	*Leucine Rich Repeat Containing 14*	14	424798	428272
RECQL4	*RecQ Like Helicase 4*	14	428460	434942
MFSD3	*Major Facilitator Superfamily Domain Containing 3*	14	435003	437076
GPT	*Glutamic--Pyruvic Transaminase*	14	438530	442163
PPP1R16A	*Protein Phosphatase 1 Regulatory Subunit 16A*	14	443246	448420
FOXH1	*Forkhead Box H1*	14	469132	470687
KIFC2	*Kinesin Family Member C2*	14	470777	478486
CYHR1	*Antisense To CYHR1*	14	478582	492655
TONSL	*Tonsoku Like, DNA Repair Protein*	14	496271	507275
VPS28	*VPS28 Subunit Of ESCRT-I*	14	508418	512819
SLC39A4	*Solute Carrier Family 39 Member 4*	14	533918	538406
CPSF1	*Cleavage And Polyadenylation Specific Factor 1*	14	542386	556837
ADCK5	*AarF Domain Containing Kinase 5*	14	556020	570498
SLC52A2	*Solute Carrier Family 52 Member 2*	14	578057	580805
FBXL6	*F-Box And Leucine Rich Repeat Protein 6*	14	580951	583957
TMEM249	*Transmembrane Protein 249*	14	584956	586597
SCRT1	*Scratch Family Transcriptional Repressor 1*	14	594540	600190
DGAT1	*Diacylglycerol O-Acyltransferase 1*	14	603813	612791
HSF1	*Heat Shock Transcription Factor 1*	14	613328	634349
BOP1	*BOP1 Ribosomal Biogenesis Factor*	14	634453	652447
SCX	*Scleraxis BHLH Transcription Factor*	14	646600	648020
MROH1	*Maestro Heat Like Repeat Family Member 1*	14	652785	702430
TSSK5	*Testis Specific Serine Kinase 4*	14	709459	712018
HGH1	*HGH1 Homolog*	14	712202	715270
WDR97	*WD Repeat Domain 97*	14	720987	723829
MAF1	*MAF1 Homolog, Negative Regulator Of RNA Polymerase III*	14	728324	732756
SHARPIN	*SHANK Associated RH Domain Interactor*	14	732964	737292
CYC1	*Cytochrome C1*	14	738124	740518
GPAA1	*Glycosylphosphatidylinositol Anchor Attachment 1*	14	750608	753850
EXOSC4	*Exosome Component 4*	14	755134	757010
OPLAH	*5-Oxoprolinase, ATP-Hydrolysing*	14	765421	774581
SMPD5	*Sphingomyelin Phosphodiesterase 5 (Pseudogene)*	14	774643	776724
SPATC1	*Spermatogenesis And Centriole Associated 1*	14	778853	806391
GRINA	*Glutamate Ionotropic Receptor NMDA Type Subunit Associated Protein 1*	14	826740	830066
PARP10	*Poly(ADP-Ribose) Polymerase Family Member 10*	14	832766	839743
PLEC	*Plectin*	14	839972	896647
EPPK1	*Epiplakin 1*	14	940689	955681
NRBP2	*Nuclear Receptor Binding Protein 2*	14	961099	968482
PUF60	*Poly(U) Binding Splicing Factor 60*	14	972914	986379
SCRIB	*Scribble Planar Cell Polarity Protein*	14	987039	1006477

IQANK1	*IQ Motif And Ankyrin Repeat Containing 1*	14	1006976	1027840
FAM83H	*Family With Sequence Similarity 83 Member H*	14	1025239	1039266
MAPK15	*Mitogen-Activated Protein Kinase 15*	14	1040716	1066536
CCDC166	*Coiled-Coil Domain Containing 166*	14	1058869	1060642
ZNF623	*Zinc Finger Protein 623*	14	1071877	1073361
GFUS	*GDP-L-Fucose Synthase*	14	1094043	1099107
PYCR3	*Pyrroline-5-Carboxylate Reductase 3*	14	1101602	1108734
TIGD5	*Tigger Transposable Element Derived 5*	14	1111425	1113356
EEF1D	*Eukaryotic Translation Elongation Factor 1 Delta*	14	1113551	1126356
NAPRT	*Nicotinate Phosphoribosyltransferase*	14	1127518	1131606
MROH6	*Maestro Heat Like Repeat Family Member 6*	14	1132386	1136717
GSDMD	*Gasdermin D*	14	1139890	1146602
ZC3H3	*Zinc Finger CCCH-Type Containing 3*	14	1153788	1215120
MAFA	*MAF BZIP Transcription Factor A*	14	1225047	1226102
KCNT2	*Potassium Sodium-Activated Channel Subfamily T Member 2*	16	6388192	6826502
CA5A	*Carbonic Anhydrase 5A*	18	13345873	13368386
BANP	*BTG3 Associated Nuclear Protein*	18	13381283	13450245
ZNF469	*Zinc Finger Protein 469*	18	13698486	13709861
ZFPM1	*Zinc Finger Protein, FOG Family Member 1*	18	13720107	13781533
ZC3H18	*Zinc Finger CCCH-Type Containing 18*	18	13834154	13877327
CYBA	*Cytochrome B-245 Alpha Chain*	18	13883779	13893147
MVD	*Mevalonate Diphosphate Decarboxylase*	18	13893902	13900564
SNAI3	*Snail Family Transcriptional Repressor 3*	18	13911498	13919763
CTU2	*Cytosolic Thiouridylase Subunit 2*	18	13919854	13939801
RNF166	*Ring Finger Protein 166*	18	13924376	13932707
PIEZO1	*Piezo Type Mechanosensitive Ion Channel Component 1 (Er Blood Group)*	18	13938926	13995874
CDT1	*Chromatin Licensing And DNA Replication Factor 1*	18	14008412	14012515
APRT	*Adenine Phosphoribosyltransferase*	18	14013051	14015824
GALNS	*Galactosamine (N-Acetyl)-6-Sulfatase*	18	14016352	14032348
TRAPPC2L	*Trafficking Protein Particle Complex Subunit 2L*	18	14032588	14038555
PABPN1L	*PABPN1 Like, Cytoplasmic*	18	14037920	14043929
CBFA2T3	*CBFA2/RUNX1 Partner Transcriptional Co-Repressor 3*	18	14051588	14127897
ACSF3	*Acyl-CoA Synthetase Family Member 3*	18	14203972	14248441
CDH15	*Cadherin 15*	18	14257853	14277025
SLC22A31	*Solute Carrier Family 22 Member 31*	18	14277507	14282248
ANKRD11	*Ankyrin Repeat Domain Containing 11*	18	14327315	14446553
SPG7	*SPG7 Matrix AAA Peptidase Subunit, Paraplegin*	18	14463306	14484611
RPL13	*Ribosomal Protein L13*	18	14490263	14492655
CPNE7	*Copine 7*	18	14500473	14512698
DPEP1	*Dipeptidase 1*	18	14525082	14541878
CHMP1A	*Charged Multivesicular Body Protein 1A*	18	14550491	14557240

SPATA33	*Spermatogenesis Associated 33*	18	14557133	14566670
CDK10	*Cyclin Dependent Kinase 10*	18	14568489	14576084
SPATA2L	*Spermatogenesis Associated 2 Like*	18	14575038	14579286
VPS9D1	*VPS9 Domain Containing 1*	18	14582685	14592543
ZNF276	*Zinc Finger Protein 276*	18	14593024	14607123
FANCA	*FA Complementation Group A*	18	14606881	14644928
SPIRE2	*Spire Type Actin Nucleation Factor 2*	18	14652352	14679143
TCF25	*Transcription Factor 25*	18	14681557	14704281
MC1R	*Melanocortin 1 Receptor*	18	14705093	14706843
TUBB3	*Tubulin Beta 3 Class III*	18	14708940	14717352
DEF8	*Differentially Expressed In FDCP 8 Homolog*	18	14722687	14736830
DBNDD1	*Dysbindin Domain Containing 1*	18	14764643	14772547
GAS8	*Growth Arrest Specific 8*	18	14772666	14793387
URAH	*Urate (Hydroxyiso-) Hydrolase, Pseudogene*	18	14792613	14795659
SHCBP1	*SHC Binding And Spindle Associated 1*	18	14881869	14919139
VPS35	*VPS35 Retromer Complex Component*	18	14984970	15012555
ORC6	*Origin Recognition Complex Subunit 6*	18	15013053	15021378
MKRN3	*Makorin Ring Finger Protein 3*	21	1160774	1163589
MAGEL2	*MAGE Family Member L2*	21	1205086	1208637
NDN	*Necdin, MAGE Family Member*	21	1252109	1253718
KHDRBS2	*KH RNA Binding Domain Containing, Signal Transduction Associated 2*	23	270838	961226
BTNL2	*Butyrophilin Like 2*	23	25858687	25883331
